# Mitral regurgitation detected during the intraoperative period after atrial septal defect closure: a case report

**DOI:** 10.1186/s13019-019-0964-1

**Published:** 2019-07-22

**Authors:** Joohyun Jun, Min-Kyung Kang, Joon-Sang Hyeon, Eunha Choi, Youngrok Kim, Ki Seok Kim, Mi Hwa Chung, In-Jung Jun

**Affiliations:** 1 Department of Anesthesiology and Pain Medicine, Kangnam Sacred Heart Hospital, Hallym University College of Medicine, 948-1, Daerim 1-dong, Yeongdeungpo-gu, Seoul, 150-950 South Korea; 2 Department of Cardiology, Kangnam Sacred Heart Hospital, Hallym University Medical Center, Seoul, South Korea; 3 Department of Anesthesiology and Pain Medicine, Fine Pain Clinic, Seochogu, Seoul, South Korea

**Keywords:** Mitral regurgitation, Atrial septal defect, Echocardiography

## Abstract

**Background:**

Atrial septal defect (ASD) is a congenital cardiac defect often diagnosed in adult patients. Mitral regurgitation (MR) observed in ASD patients mostly improves after ASD closure. However, a subset of adult ASD patients present new-onset MR or aggravation of preexisting MR after ASD closure. Intraoperative MR aggravation after surgical ASD closure is a rare occurrence which has not been reported in the literature to date.

**Case presentation:**

A 54-year-old woman was referred to our center due to large secundum ASD with a diameter of 17 mm which was incidentally detected on pre-operative echocardiography at a local clinic. Surgical repair of ASD under mini-thoracotomy was performed. After completion of the operation, intra-operative transesophageal echocardiography showed newly developed Grade II MR which subsequently deteriorated to severe level on postoperative day 3. Because the patient was asymptomatic, we decided to observe closely and treat conservatively with diuretics. Thereafter, echocardiography was evaluated on postoperative day 10 and MR disappeared to trivial level.

**Conclusions:**

Intraoperative MR aggravation is a rare complication following ASD closure. The possibility of MR aggravation should be evaluated in all ASD patients prior to surgery. This case highlights the importance of mitral leaflet examination after ASD closure for early detection of MR.

## Background

Atrial septal defect (ASD) is a congenital cardiac defect often diagnosed in adult patients [1]. Mild mitral regurgitation (MR) associated with ASD is often improved after shunt closure which reduces right ventricular volume overload and restores ventricular septal configuration [2]. However, a subset of adult ASD patients with coexisting structural changes in the heart chambers present new-onset MR or aggravation of preexisting MR after ASD closure [3]. There are several reports of postoperative MR aggravation after ASD closure [3–5], but to the best of our knowledge, intraoperative detection of increased MR has not been reported previously. Here, we evaluate a rare case of MR aggravation which was detected during the intraoperative period directly after ASD closure.

## Case presentation

**Fig. 1:**
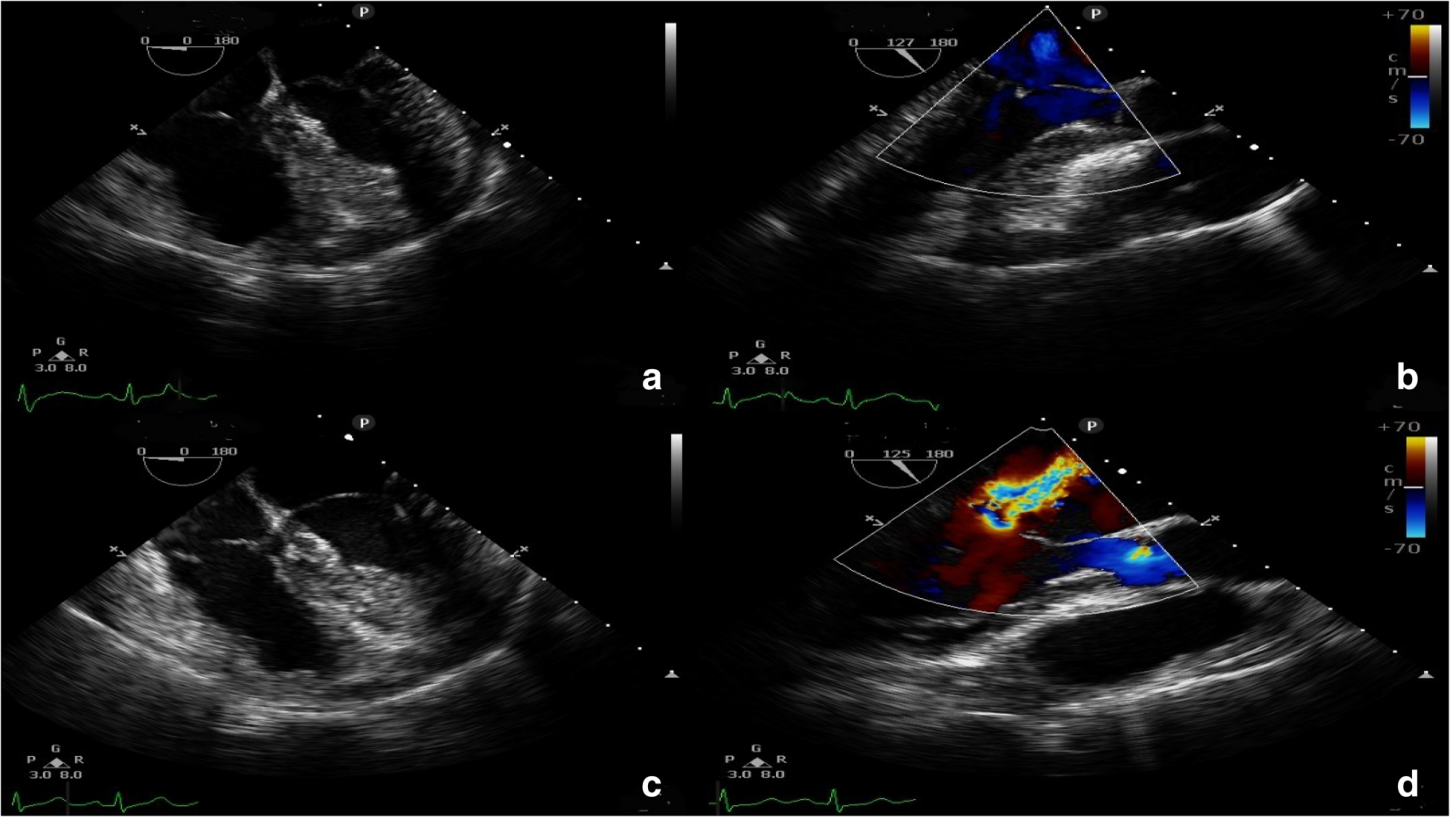
Transesophageal echocardiography was evaluated before (**a**,**b**) and after (c,d) ASD closure. (**a**) Preoperative D-shaped small left ventricle with dilated right ventricle. (**b**) No mitral regurgitation noted. (**c**) Postoperative rightward deviated ventricular septum with dilated mitral valve annulus. (**d**) Newly developed mitral regurgitation after atrial septal defect closure

A 54-year-old woman was referred to our center due to right ventricular enlargement which was incidentally detected on pre-operative echocardiography for ankle surgery at a local clinic. The patient was asymptomatic and in normal sinus rhythm. A transthoracic echocardiography (TTE) showed a large secundum ASD with a diameter of 17 mm. A transesophageal echocardiography (TEE) was performed and showed 20 × 23 mm secundum ASD with left to right shunt and right ventricle (RV), right atrium (RA) enlargement (Fig. 1a). The patient had a D-shaped small left ventricle (LV) with a left ventricular ejection fraction of 59%. Mitral valve leaflets were normal with no MR detected (Fig. 1b). Moderate tricuspid regurgitation (Grade II) due to dilated tricuspid valve annulus (46 mm) and mild pulmonary hypertension were observed. The rims to both sides of the superior vena cava and inferior vena cava were short, thus surgical repair of ASD under mini-thoracotomy was planned.

In the operating room, standard vital signs (pulse oximetry, end-tidal carbon dioxide, electrocardiogram, and non-invasive blood pressure) were monitored. The left radial artery was catheterized for continuous arterial blood pressure monitoring. After 3 min of 100% pre-oxygenation, general anesthesia was induced with midazolam (3 mg) followed by continuous infusion of propofol with remifentanil, and bolus administration of rocuronium (50 mg). The patient was intubated with a 35 Fr left-sided double-lumen tube for one-lung ventilation. A central venous catheter was inserted via the right subclavian vein because the right internal jugular vein was reserved for superior vena cava cannulation for cardiopulmonary bypass (CPB). A TEE probe was inserted to permit close observation.

Right anterolateral mini-thoracotomy was done via 4th Intercostal space. Following full anticoagulation with heparin given at a dose of 300 IU/kg, CPB was instituted using femoral artery, femoral vein and right internal jugular vein cannulation. Next, the aortic Detachable Glauber clamp (Cardiomedical GmbH, Germany) was deployed for aortic cross-clamp, and 2000 mL of Custodiol® HTK (Koehler Chemie, Bensheim, Germany) solution was infused through aortic root cannula for myocardial protection. Moderate Hypothermia of 31.5 °C was permitted as measured by nasopharyngeal and rectal probes. Subsequently, right atrium was opened and ASD was closed with a trimmed bovine pericardial patch. Tricuspid ring annuloplasty and right atrium reduction plasty were also conducted. After completion of the operation, right atrium was closed and CPB was weaned.

Intra-operative TEE showed that ASD was closed with no remnant inter-atrial shunt. There was no tricuspid regurgitation and left ventricular ejection fraction was 55%. Newly developed Grade II MR with end-diastolic rightward deviated inter-ventricular septum was detected which was not found in pre-operative echocardiography (Fig. 1c, d). We notified the surgeon of the newly developed MR. Because no abnormal findings, such as mitral valve prolapse, perforation, or chordae rupture, were observed in the mitral valve leaflets, it was determined that the surgery should proceed. No further adverse surgical events occurred throughout the remainder of the surgical procedure. The surgery lasted for 345 min with the CPB time of 190 min and aortic cross-clamp time of 140 min. The estimated blood loss of 800 ml. After surgery, the patient was transferred to the Intensive Care Unit. Bilateral lung haziness due to acute MR was observed in the immediate post-operative chest x-ray. Otherwise, the vital signs were stable without complaint of any symptoms. The patient was extubated after 3 h on arrival of the intensive care unit and transferred to general ward on postoperative day (POD) 1.

**Fig. 2:**
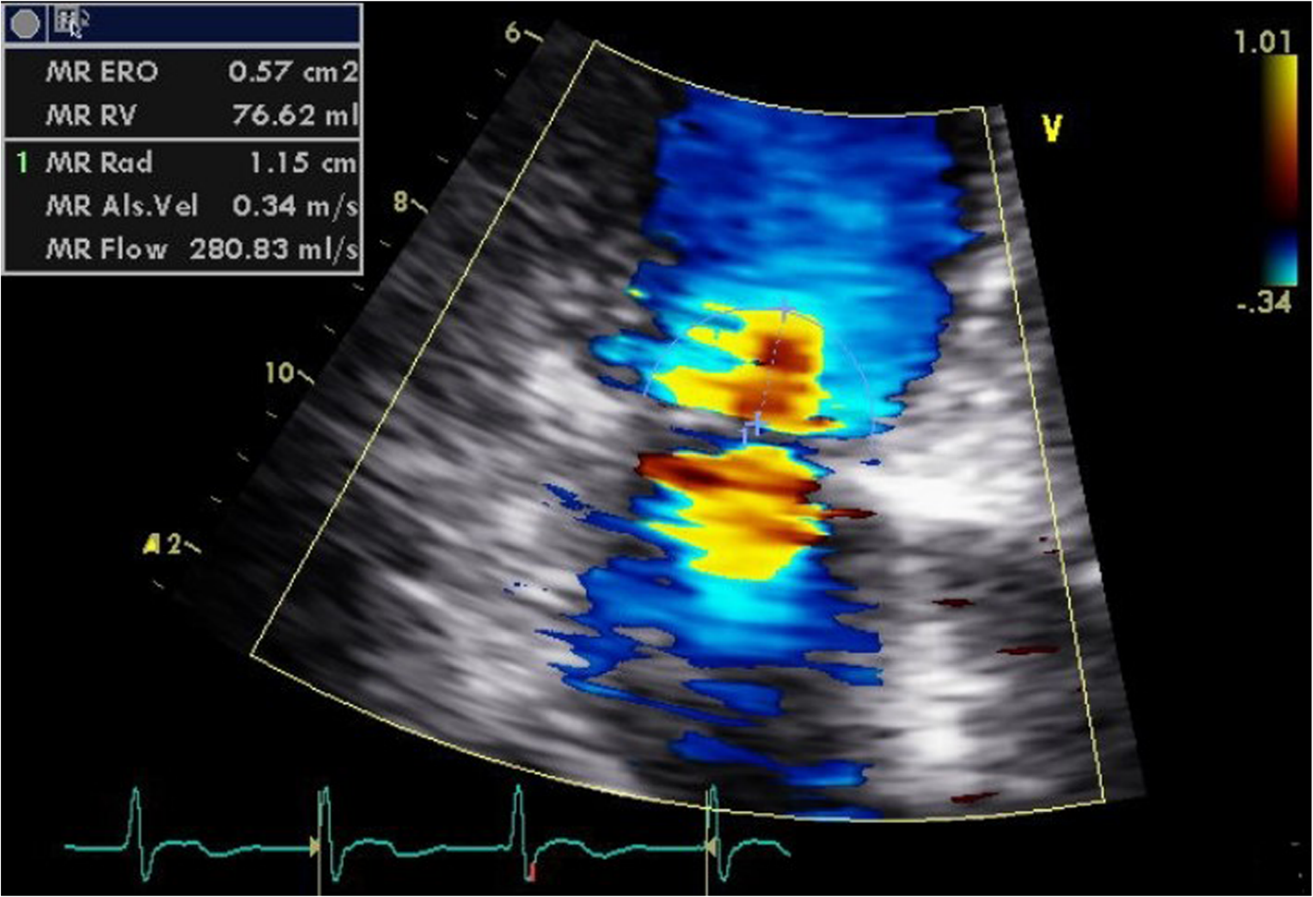
Transthoracic echocardiography showed that mitral regurgitation was deteriorated to a severe level on postoperative day 3 (Effective regurgitation orifice area 0.57 cm^2^)

**Fig. 3:**
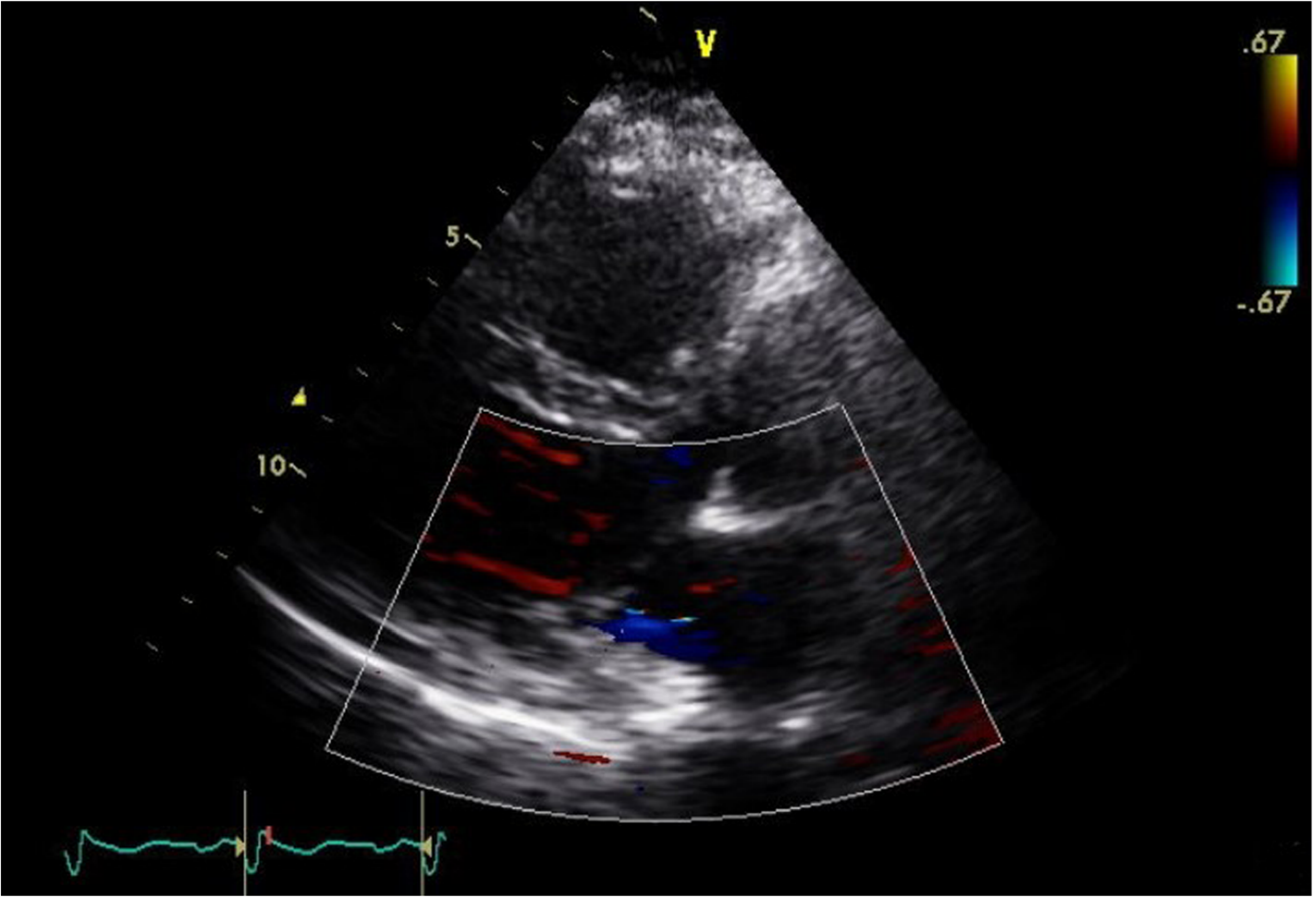
On postoperative day 10, transthoracic echocardiography showed that mitral regurgitation disappeared

Transesophageal echocardiography on POD 3 confirmed that the ASD patch was intact without shunt flow or remnant tricuspid regurgitation. Both left and right ventricular function was well preserved with left ventricular ejection fraction of 69%. However, LV diastolic dysfunction (E/E’ = 26) and aggravated pulmonary hypertension which was not observed in the preoperative TEE was found. The MR was shown to have deteriorated to severe level without evidence of vegetation or chordae rupture (Fig. 2). Because the patient was asymptomatic, conservative treatment using diuretics and close monitoring was determined to be the best course of action. Daily follow-up chest x-ray showed gradual improvement in pulmonary edema. On POD 6, the patient was discharged and attend follow-up outpatient appointments. On POD 10, TTE was evaluated. MR disappeared to trivial level and the LV chamber size and deviated septum became normalized (Fig. 3).

## Discussion and conclusions

Mitral regurgitation is observed in less than 10% of adult ASD patients caused by mitral valve prolapse related to longstanding structural changes of the LV secondary to RV volume overload [1]. Generally, ASD closure improves MR by reducing the RV volume [4]. However, deterioration of mitral valve competence is reported in around 10% of patients undergoing ASD closure [3–5].

Previous studies have suggested several mechanisms of aggravation of MR [3, 4]. Intrinsic MV abnormality and subsequent poor coaptation of MV may have been masked by the abnormal septal configuration in ASD patients [6]. Also, perioperative atrial fibrillation is related to aggravation of MR [1]. Atrial fibrillation frequently causes left atrium (LA) enlargement and atrial fibrillation patients with normal LA size often have impaired LA function [7, 8]. With LA enlargement or LA impairment, LA posterior wall extends beyond LV and results in poor coaptation of mitral valve [1, 9].

Our patient had no mitral lesion and presented normal electrocardiography. Thus, the mechanism of increased MR of the present case is assumed as longstanding adaptation of MV annulus geometry to the abnormal septal configuration and failure of adaptation to the cardiac geometric changes after ASD closure [10]. ASD shunt closure increases left ventricular volume and leftward deviated interventricular septum becomes normal [11]. Although mitral configuration normalizes in most of the patients, failure of mitral competence to acutely improved LV geometry after ASD closure may cause worsening of MR [10]. In a subset of adult ASD patients with underlying age-dependent decline of LV compliance, the possibility of failure of mitral competence to acutely improved LV geometry becomes higher [2, 12].

Preoperative echocardiographic features are useful in predicting postoperative MR. Risk factors for MR include D-shaped LV, large ASD, small LV and RV horizontal diameter to vertical diameter ratio, abnormal shape of mitral leaflets [1, 2, 4]. Also, larger pulmonary to systemic flow ratio (Qp/Qs) and high peak tricuspid regurgitation velocity have been identified as risk factors for MR [13]. As our patient had preoperative echocardiographic features of D-shaped LV, large ASD, and small LV horizontal diameter to vertical diameter ratio, the risk of postoperative MR after ASD was high.

The present case is unique in that MR aggravation was detected during the intraoperative period directly after ASD closure and the MV apparatus quickly adapted LV geometry by POD 10. We believe that intraoperative MR aggravation after surgical ASD closure is a rare occurrence which, to the best of our knowledge, has not been reported in the literature to date. Cardiac remodeling usually begins immediately after ASD closure and continues to steadily improve over subsequent months [14, 15]. MR worsening is generally detected within 5 years in 50–62.5% of patients and the rest in more than 5 years after ASD closure [1]. Surgical closure, compared to catheter closure, may have detrimental effect on diastolic function in the early postoperative period from use of CPB which may result in MR worsening [14]. We excluded myocardial stunning as a factor of postoperative mitral regurgitation aggravation because both left and right ventricular function was well preserved without need of inotropic support. We assumed that acute LV volume loading after ASD shunt closure in the preexisting D-shaped small LV configuration influenced the development of severe MR. Moreover, acute diastolic dysfunction appear to have contributed in MR aggravation by increasing LV filling pressure [16]. Acute compensation may have been possible because the patient did not have left sided heart failure and mitral leaflets were normal. Thus, LV compliance improved promptly inducing normalization of cardiac chambers with postoperative supportive care only.

For patients at high-risk of MR deterioration, prophylactic mitral valve annuloplasty may be considered [4]. We decided to observe closely and treat conservatively with diuretics instead of conducting mitral annuloplasty. We reached the decision because mitral leaflets were normal and we believed that reopening the heart chamber after the mini-thoracotomy placed an unnecessary burden on the patient. Mitral leaflet examination through intraoperative echocardiography is crucial for early detection of MR. The American College of Cardiology/American Heart Association guidelines for the management of adults with congenital heart disease recommend clinical and transesophageal echocardiographic follow-up after ASD closure at 24 h, 1 month, 6 months, and 1 year and at regular intervals there after [17]. If severe symptomatic MR develops with mitral leaflet motion abnormality, surgery may be advised.

## Abbreviations

ASD: atrial septal defect
CPB: cardiopulmonary bypass
LA: left atrium
LV: left ventricle
MR: mitral regurgitation
POD: postoperative day
RA: right atrium
RV: right ventricle
TEE: transesophageal echocardiography
TTE: transthoracic echocardiography
