# Correction to: Mitral regurgitation detected during the intraoperative period after atrial septal defect closure: a case report

**DOI:** 10.1186/s13019-019-0995-7

**Published:** 2019-10-21

**Authors:** Joo Hyun Jun, Min-Kyung Kang, Joon-Sang Hyeon, Eunha Choi, Youngrok Kim, Ki Seok Kim, Mi Hwa Chung, In-Jung Jun

**Affiliations:** 1 Department of Anesthesiology and Pain Medicine, Kangnam Sacred Heart Hospital, Hallym University College of Medicine, 948-1, Daerim 1-dong, Yeongdeungpo-gu, Seoul, 150-950 South Korea; 2 Department of Cardiology, Kangnam Sacred Heart Hospital, Hallym University Medical Center, Seoul, South Korea; 3 Department of Anesthesiology and Pain Medicine, Fine Pain Clinic, Seochogu, Seoul, South Korea

**Correction to: J Cardiothorac Surg (2019) 14:140**

**https://doi.org/10.1186/s13019-019-0964-1**

The original article [1] contained a typo in author, Joo Hyun Jun’s name. This has now been corrected.
